# Survival and Growth of Epiphytic Ferns Depend on Resource Sharing

**DOI:** 10.3389/fpls.2016.00416

**Published:** 2016-03-31

**Authors:** Hua-Zheng Lu, Liang Song, Wen-Yao Liu, Xing-Liang Xu, Yue-Hua Hu, Xian-Meng Shi, Su Li, Wen-Zhang Ma, Yan-Fen Chang, Ze-Xin Fan, Shu-Gang Lu, Yi Wu, Fei-Hai Yu

**Affiliations:** ^1^Key Laboratory of Tropical Forest Ecology, Xishuangbanna Tropical Botanical Garden, Chinese Academy of SciencesMengla, China; ^2^University of the Chinese Academy of SciencesBeijing, China; ^3^Key Laboratory of Ecosystem Network Observation and Modeling, Institute of Geographic Sciences and Natural Resources Research, Chinese Academy of SciencesBeijing, China; ^4^Kunming Institute of Botany, Chinese Academy of SciencesKunming, China; ^5^Institute of Ecology and Geobotany, Yunnan UniversityKunming, China; ^6^School of Nature Conservation, Beijing Forestry UniversityBeijing, China

**Keywords:** canopy-dwelling plants, clonal growth, clonal integration, forest canopy, habitat adaptation, montane moist forest, physiological integration

## Abstract

Locally available resources can be shared within clonal plant systems through physiological integration, thus enhancing their survival and growth. Most epiphytes exhibit clonal growth habit, but few studies have tested effects of physiological integration (resource sharing) on survival and growth of epiphytes and whether such effects vary with species. We conducted two experiments, one on individuals (single ramets) and another on groups (several ramets within a plot), with severed and intact rhizome treatments (without and with physiological integration) on two dominant epiphytic ferns (*Polypodiodes subamoena* and *Lepisorus scolopendrium*) in a subtropical montane moist forest in Southwest China. Rhizome severing (preventing integration) significantly reduced ramet survival in the individual experiment and number of surviving ramets in the group experiment, and it also decreased biomass of both species in both experiments. However, the magnitude of such integration effects did not vary significantly between the two species. We conclude that resource sharing may be a general strategy for clonal epiphytes to adapt to forest canopies where resources are limited and heterogeneously distributed in space and time.

## Introduction

Environments are characterized by patchy distributions of abiotic and biotic factors (Alpert and Mooney, 1996; Chen et al., 2002; Jahnke et al., 2015). Clonal plants can integrate information about such environmental heterogeneity and respond accordingly (Louâpre et al., 2012; Wang et al., 2013; Oborny and Hubai, 2014; Chen et al., 2015; Saunders and Pezeshki, 2015). One strategy by which clonal plants cope with environmental heterogeneity is physiological integration, i.e., the capacity to share resources among interconnected ramets (Hutchings and Wijesinghe, 1997; Herben and Suzuki, 2001; Song et al., 2013; Roiloa et al., 2014; Dong et al., 2015). Physiological integration enables parent ramets to support offspring ramets (Matlaga and Sternberg, 2009; Oborny and Hubai, 2014; Roiloa et al., 2014; Glover et al., 2015) and ramets growing in favorable conditions to support those in unfavorable conditions (Roiloa et al., 2007; Xu L. et al., 2012; Kui et al., 2013; Tuya et al., 2013; Cornelissen et al., 2014; Luo et al., 2014).

Forest canopies house ca. 50% of terrestrial biodiversity (Ozanne et al., 2003; May, 2010; Lowman and Schowalter, 2012). As a key component of tropical and subtropical floras (Benzing, 2012; Zotz, 2013), canopy-dwelling epiphytes serve important ecological functions in forest hydrology and nutrient fluxes (Umana and Wanek, 2010; Zhang et al., 2015). However, epiphytic habitats are usually described as “harsh” because tree crowns are characterized by a limited storage capacity for available nutrients and water, sporadic and dilute nutrient inputs, low physical stability, extreme fluctuations in moisture and temperature, high wind speed, and severe and variable vapor pressure deficits (Théry, 2001; Zotz and Hietz, 2001; Benzing, 2012; Lowman and Schowalter, 2012). Significant variation in resource availability can occur at small spatial and temporal scales, and short-term drought can occur even in wet seasons of tropical rain forests (Zotz and Hietz, 2001; Watkins et al., 2007a). How epiphytes adapt to the harsh and heterogeneous environments of forest canopies remains one of the most fascinating questions in plant ecology (Benzing, 2012; Lowman and Schowalter, 2012; Reyes-García et al., 2012).

Almost all epiphytic bryophytes and lichens and many vascular epiphytes are capable of clonal growth (Jackson et al., 1985; During, 1990; de Kroon and van Groenendael, 1997; Benzing, 2012; Robinson and Miller, 2013). Different ramets within a clone are often interconnected via rhizomes, stolons or roots so that resource sharing (physiological integration) within the clone is possible (Eilts et al., 2011; Cornelissen et al., 2014; Weiser and Smycka, 2015). In the past decades, roles of physiological integration have been extensively documented in different species and in different habitats (Jackson et al., 1985; de Kroon and van Groenendael, 1997; Song et al., 2013). However, little is known about how physiological integration facilitates adaptation of epiphytes to forest canopies.

Recently, we selected one clonal, facultative, epiphytic fern to test effects of physiological integration in both epiphytic and terrestrial habitats in the dry season in a subtropical montane moist forest (Lu et al., 2015). We found that clonal integration contributed greatly to survival and growth of this species, and that the effect was more important in forest canopies than in forest understories (Lu et al., 2015). However, the target species possesses the unique aspects of facultative epiphytes and overwintering leaves (Lu et al., 2015), and is a common yet not dominant species in the forest. Furthermore, the experiment was carried out during the dry season when seasonal drought occurred, whereas most dominant epiphytes stop growing (shed leaves) in the dry season. Thus, it is still unknown whether clonal integration also plays an important role in dominant epiphytes and during the wet season. We hypothesize that (1) physiological integration can also increase survival and growth of dominant clonal epiphytes in the wet season.

In subtropical montane moist forests in Southwest China, eight of the nine dominant vascular epiphytes are ferns (Supplementary Table 1). Seven of these ferns produce long, creeping rhizomes that may potentially be investigated in the wet season (Xu and Liu, 2005; Ma, 2009). Because epiphytic ferns vary in morphology, physiology and phenology (Schneider et al., 2004; Watkins et al., 2007b), it is likely that these epiphytic species have adapted to habitats using various strategies. We thus hypothesize that (2) clonal epiphytes with divergent traits differ in their degree of dependence on clonal integration.

To test the hypotheses, we conducted two field experiments on two dominant epiphytes with divergent traits in a wet season in a subtropical montane moist forest in Southwest China. Specifically, we addressed two questions. (1) Does clonal integration increase survival and growth of dominant epiphytes during the wet season when water stress was seemingly weak? (2) If it does, does the effect of clonal integration on survival and growth differ between the two epiphytes with divergent traits? By addressing such questions in two dominant epiphytes and in growing (wet) seasons, we aim to test whether clonal integration is a general strategy for clonal epiphytes to adapt to forest canopies. The results obtained will deepen our understanding of the strategies of epiphytes dwelling on forest canopies.

## Materials and methods

### Study site

We conducted the two field experiments in a primary subtropical montane moist forest in the Xujiaba region (24° 32′ N, 101° 01′ E) of Yunnan province, China, a core area covering 5100 ha of the northern crest of the Ailao Mountain National Nature Reserve. In this region, water loss occurs during the dry season, while water accumulates during the wet season (You et al., 2013a; Lu et al., 2015). During 2000–2010, the mean annual precipitation was 1874 mm, with 87% occurring in the wet season (May to October) and 13% in the dry season (November to April), the mean annual relative humidity was 84%, and the mean air temperature was 11.1°C (5.6°C in January and 15.3°C in July; Song et al., 2012). The forest is dominated by *Lithocarpus xylocarpus, Castanopsis wattii, L. chintungensis, Schima noronhae, Machilus viridis*, and *Hartia sinensis*, and also inhabited by a diverse community of epiphytes (Li et al., 2014).

### Target species

*Polypodiodes subamoena* (C. B. Clarke) Ching and *Lepisorus scolopendrium* (Ham. ex. D. Don) Menhra are two dominant vascular epiphytes in the montane moist forest (Xu and Liu, 2005; Ma, 2009). They mainly inhabit tree bark, junctions or rocks, and are capable of clonal growth via long, creeping rhizomes with adventitious roots (Zhang, 2012). The fronds of both ferns wither in the dry season, but their rhizomes can persist for several years. These two ferns exhibit different functional traits (i.e., morphology, physiology, and growth; Table 1). *P. subamoena* bears remote compound fronds and pinnatipartite (15–20 paired), herbaceous laminas and mainly occurs at 2400–3300 m a.s.l., whereas *L. scolopendrium* bears a close single frond and a herbaceous or papery lamina and occurs at 500–2800 m a.s.l. (Zhang, 2012).

**Table 1:** **Contrasting functional traits of the ramets of two species, *Polypodiodes subamoena* and *Lepisorus scolopendrium***.

**Trait**	***P. subamoena***	***L. scolopendrium***	***t***	***P***
Frond length (cm)	19.73 ± 0.74	16.14 ± 0.85	3.2	**0.002**
Frond width (cm)	4.61 ± 0.22	2.54 ± 0.10	8.7	**<0.001**
Frond thickness (mm)	0.33 ± 0.02	0.99 ± 0.04	−14.1	**<0.001**
F_v_/F_m_	0.74 ± 0.01	0.79 ± 0.01	−3.4	**0.001**
Aboveground mass per ramet (g)	0.24 ± 0.02	0.12 ± 0.01	5.6	**<0.001**
Belowground mass per ramet (g)	0.28 ± 0.02	0.25 ± 0.02	1.1	0.275
Total mass per ramet (g)	0.52 ± 0.04	0.37 ± 0.02	3.5	**0.001**
Ramet density (no. dm^−2^)	3.40 ± 0.11	6.50 ± 0.26	−11.1	**<0.001**

*The given are mean ± SE of each species and results of t-tests*.

*Bold letters in column of “P” mean significant*.

### Experiment design

#### Individual experiment

For each of the two species, we selected 60 mature ramets from the boles or crowns of 20 host trees (i.e., phorophytes) with diameter at breast height exceeding 30 cm. Ramet height of *P. subamoena* was 30.0 ± 0.4 cm (mean ± *SE*, ranging from 24.0 to 33.9 cm), and that of *L. scolopendrium* was 18.2 ± 0.3 cm (mean ± *SE*, ranging from 15.0 to 22.2 cm). Half of the ramets were randomly assigned to the severed-rhizome treatment and the other half to the intact-rhizome treatment. For the severed treatment, the rhizome internodes at both ends of the ramet were carefully exposed and cut off halfway from the ramet to prevent clonal integration. For the intact treatment, the rhizome internodes of the ramet were also carefully exposed, but no cutting was conducted so that physiological integration was allowed. The experiment started on July 26, 2013 and ended on October 26, 2013. At the end of the experiment, the survival status of all ramets was noted and the surviving ramets were harvested. A ramet was considered dead if all its fronds were shed, dried or withered. We measured frond length and width of the ramets with a ruler and frond thickness with calipers. Biomass was measured after drying the ramets at 70°C for 48 h. One day before harvest, we also measured maximum quantum yield of PS II (F_v_/F_m_) using a portable fluorometer (FSM-2; Hansatech, Norfolk, UK).

#### Group experiment

For each species, we selected 20 plots, each with at least three ramets of the target species. Plots were located on 20 phorophytes (with diameter at breast height >30 cm). Half of the plots were randomly selected and subjected to the severed-rhizome treatment, and the remaining half to the intact-rhizome treatment. For the severed treatment, the rhizomes along the edges of each plot were carefully exposed by removing surrounding soil, humus, mosses and/or lichens, if any, and cut off with a sharp blade so that ramets inside the plot were disconnected from those outside the plot to prevent integration. For the intact treatment, the rhizomes along the edges of each plot were also carefully exposed, but were kept intact (i.e., not cut off) so that ramets inside the plot were connected with those outside to allow integration. The experiment started on July 30, 2013 and ended on October 30, 2013. At harvest, we counted number of surviving ramets and measured length, width, and thickness of the fronds of each surviving ramet in each plot. One day before harvest, we measured F_v_/F_m_ using the FMS-2 on the fronds of two ramets in each plot. Biomass in each plot was measured after drying the plant materials at 70°C for 48 h.

### Statistical analyses

We analyzed the data from the two experiments separately. For the individual experiment, we used logistic regression to test the effect of rhizome severing (intact vs. severed) on survival of the ramets because the data of survival were binary (alive or dead) (McCullagh and Nelder, 1989). We used two-way ANOVA to test the effects of rhizome severing, species, and their interaction on growth (total biomass, aboveground, and belowground biomass), morphology (frond length, width and thickness), and physiology (F_v_/F_m_) of the individual ramets.

For the group experiment, we expressed the final biomass data on a per initial ramet basis because initial number of ramets differed greatly between the two species [*P. subamoena* vs. *L. scolopendrium*: 3.4 ± 0.11 vs. 6.5 ± 0.26 g (mean ± SE); *t* = −11.07, *P* < 0.001, *n* = 40]. We also calculated mean frond length, width and thickness and F_v_/F_m_ of the ramets in each plot. We then used two-way ANOVA to test the effects of rhizome severing, species and their interactions on number of surviving ramets, growth, morphology and physiology in the group experiment. When needed, data were transformed to square root or natural logarithm to meet the ANOVA assumptions. Statistical analyses were carried out with SPSS 19.0 (IBM, Armonk, NY, USA) and R software (R Development Core Team, 2012).

## Results

### Individual experiment

**Figure 1:**
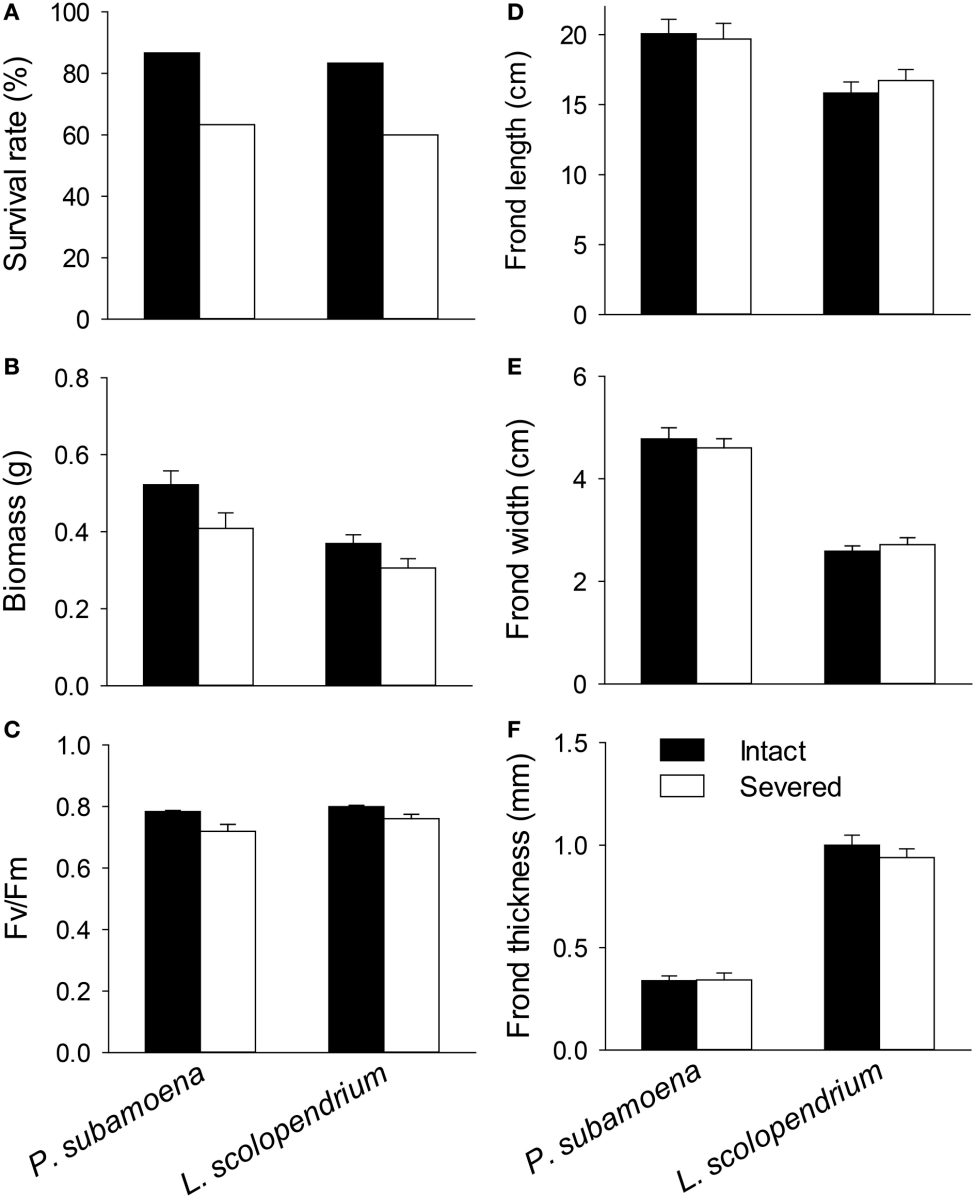
**Effects of rhizome severing on (A) survival, (B) biomass, (C) F_v_/F_m_, and (D–F) frond morphology of the two epiphytic ferns in the individual experiment**. Error bars represent SEs.

In the individual experiment, rhizome severing significantly affected survival of the single ramets (χ^2^ = 8.61, *P* = 0.003), and such effects were not species-dependent (i.e., no interaction effect; χ^2^ = 0.02, *P* = 0.893). Survival rates of the single ramets were 86.7% for *P. subamoena* and 83.3% for *L. scolopendrium* when the rhizomes were intact, but were reduced to 63.3 and 60.0% when the rhizomes were severed (Figure 1A).

Rhizome severing significantly decreased total and belowground biomass (Table 2; Figure 1B) and maximum quantum yield of PS II (F_v_/F_m_; Table 2; Figure C) of the single ramets of both epiphytes. Such effects did not depend on species (no Se × Sp interaction; Table 2). Severing had no effect on frond length, width or thickness of the single ramets of either species (Figures 1D–F). Species significantly affected biomass, F_v_/F_m_, frond length, width and thickness (Table 2; Figure 1), affirming the contrasting growth, physiological and morphological traits of these two species (Table 1).

**Table 2:** **Individual experiment results of a two-way ANOVA for effects of species and rhizome severing on biomass, F_v_/F_m_, and frond morphology**.

**Trait**	**Species (Sp)**	**Severing (Se)**	**Se × Sp**
Total mass^a^	13.18^***^	8.68^**^	0.17
Aboveground mass^a^	64.01^***^	0.74	0.58
Belowground mass	0.01	12.65^**^	2.06
F_v_/F_m_^a^	5.47^*^	17.84^***^	1.05
Frond length	15.288^***^	0.08	0.48
Frond width^a^	141.98^***^	0.03	0.61
Frond thickness	251.74^***^	0.53	0.67

F statistics are shown with significance levels (

*** *P < 0.001*;

** *P < 0.01*;

* *P < 0.05)*.

a *Analysis performed on square-root transformed data*.

### Group experiment

**Figure 2:**
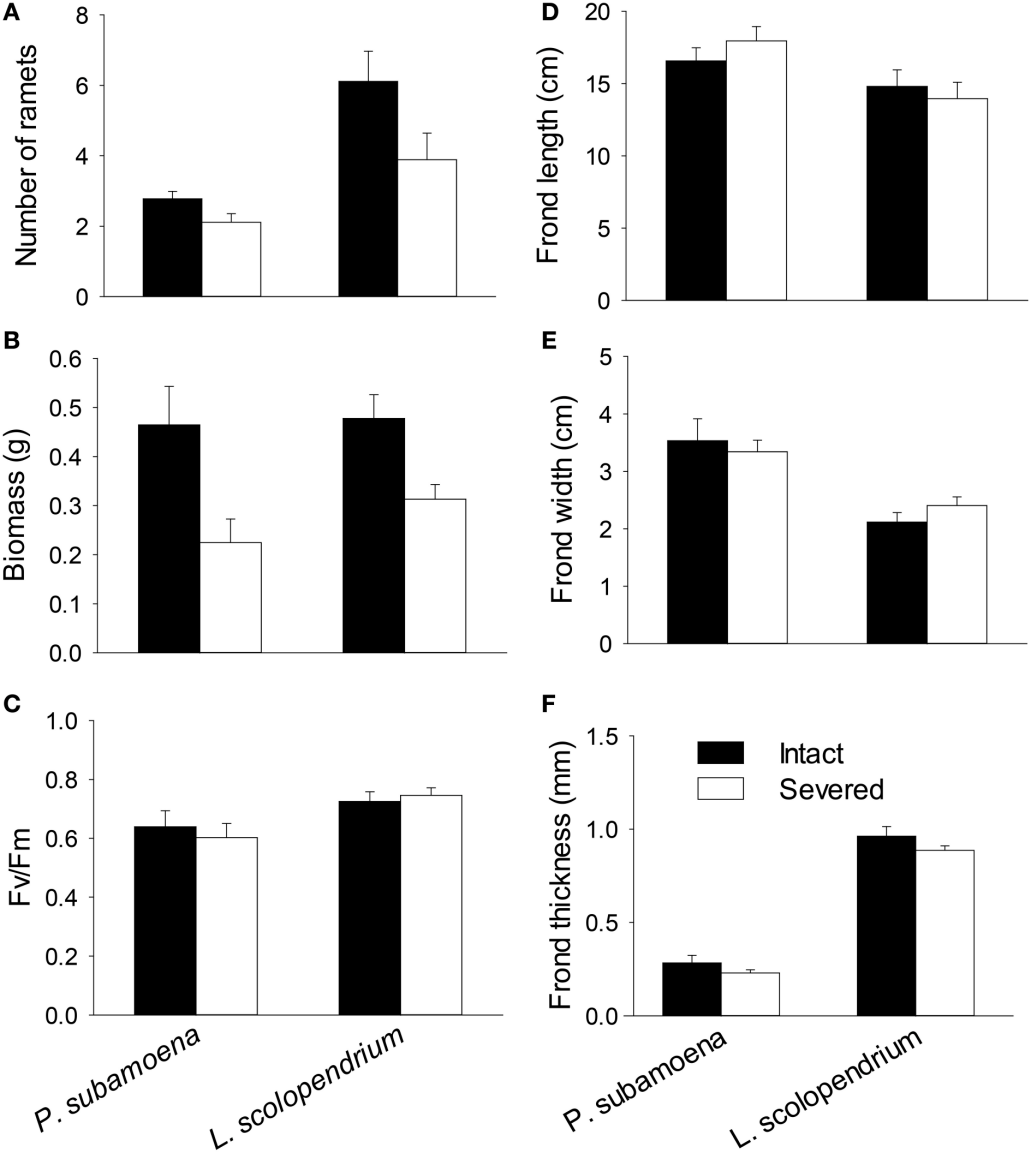
**Effects of rhizome severing on (A) ramet number, (B) biomass, (C) F_v_/F_m_, (D–F) and frond morphology of the two epiphytic ferns in the group experiment**. Error bars represent SEs.

In the group experiment, rhizome severing significantly reduced number of ramets, total biomass and belowground biomass of both epiphytes, and such effects did not depend on species (no Se × Sp interaction; Table 3; Figures 2A,B). Rhizome severing did not significantly affect F_v_/F_m_, frond length, width or thickness of either species (Table 3; Figures 2C–F). Species significantly affected aboveground and belowground biomass, F_v_/F_m_, frond length, width and thickness (Table 3; Figure 2).

**Table 3:** **Group experiment results of a two-way ANOVA for effects of species and rhizome severing on the number of ramets, biomass, F_v_/F_m_, and frond morphology**.

**Trait**	**Species (Sp)**	**Severing (Se)**	**Se × Sp**
Number of ramets^a^	0.72	6.36^*^	1.73
Total mass	0.88	13.99^**^	0.49
Aboveground mass	11.45^**^	2.39	1.55
Belowground mass^b^	4.77^*^	12.75^**^	0.12
F_v_/F_m_^b^	6.78^*^	0.04	0.43
Frond length	4.51^*^	0.04	0.67
Frond width	20.86^***^	0.03	0.87
Frond thickness^b^	308.86^***^	2.89	0.08

F statistics are shown with significance levels (

*** *P < 0.001*;

** *P < 0.01*;

* *P < 0.05)*.

a *Analysis performed on log transformed data*.

b *Analysis performed on square-root transformed data*.

## Discussion

Both individual and group experiments showed that severing rhizomes decreased survival and growth of the two dominant epiphytic ferns in the wet season, supporting the first hypothesis that clonal integration (resource sharing) contributes to performance of epiphytes. These results agree with the findings on the facultative epiphytic fern *Selliguea griffithiana* (i.e., growing in both epiphytic and terrestrial habitats) conducted in a dry season in the same forest using similar approaches (Lu et al., 2015) and also those on the terrestrial fern *Diplopterygium glaucum* in a subtropical evergreen forest in China (Du et al., 2010). While numerous studies have tested effects of clonal integration (Song et al., 2013; Glover et al., 2015; Weiser and Smycka, 2015), very few have examined those on performance of epiphytes (Lu et al., 2015). This study of multiple species verified the key role of resource sharing for epiphytes in surviving and growing in the wet season.

Extraordinary heterogeneity is present because light intensity and temperature diminish downward through the forest canopy, whereas humidity and nutrients increase toward the ground (Benzing, 2012). Epiphytes also suffer from water shortage between rainfall events even in wet seasons in tropical forests (Watkins et al., 2007a; Bartels and Chen, 2012). Our study site is characterized by a seasonal climate with variation in precipitation (You et al., 2013a). Although the forest is exposed to frequent rain and mist during the wet season, alternating wet and dry events occur daily and weekly (You et al., 2013a,b). Large trees have great microhabitat heterogeneity within their crowns and exhibit substantial changes from the inner to the outer crown in branch diameter, canopy humus cover, openness, and mean daily maximum vapor pressure deficits (Woods et al., 2015). Epiphytes dwelling in these large treetops must cope with microhabitat heterogeneity (Théry, 2001; Zotz and Hietz, 2001; Benzing, 2012). The findings of this study and the previous one (Lu et al., 2015) suggest that clonal epiphytes may evolve a high degree of clonal integration to alleviate resource stress in both wet and dry seasons. This may especially be the case for epiphytic ferns that exhibit poor water conservation owing to their limited hydraulic conductance and passive stomatal control (McAdam and Brodribb, 2012a,b).

Effects of clonal integration may differ among species (Song et al., 2013; Isogimi et al., 2014) and even among genotypes of the same species (Alpert et al., 2003; D'Hertefeldt et al., 2014; Zhou et al., 2014). For instance, rhizomatous species may be more reliant on clonal integration than stoloniferous species (Song et al., 2013), and genotypes from sand dunes have shown a greater impact of clonal integration than those from grasslands (Alpert, 1999). Although, the two epiphytic ferns differ greatly in morphological, physiological, and growth traits (Tables 1–3, Figures 1, 2), we found that the effects of clonal integration on ramet survival or growth did not differ significantly between the two epiphytes. These results thus do not support the second hypothesis, and suggest that clonal integration may be a general strategy for clonal epiphytes to survive and grow in forest canopies where resources are rather limited and also heterogeneously distributed in space and in time.

Clonal integration had a significant effect on F_v_/F_m_ of epiphytes in the individual experiment, but not in the group experiment (Tables 2, 3, Figures 1, 2). Previous studies also showed contrasting effects of clonal integration on photochemical activity of ramets (Luo et al., 2014; Roiloa et al., 2014). For instance, integration significantly affected photochemical activity of *Alternanthera philoxeroides* (Luo et al., 2014) and *Fragaria vesca* (Roiloa et al., 2014), but had little effect on that of the terrestrial fern *D. glaucum* (Du et al., 2010). Thus, effects of clonal integration on photochemical activity of the fronds may not be translated into the effects on survival and growth of the ramets. Data on survival and growth are more robust to evaluate the benefits of clonal integration.

We observed little impact of clonal integration on frond morphology of either of the epiphytes in either of the experiments (Tables 2, 3, Figures 1, 2), agreeing with the findings of our previous study (Lu et al., 2015). However, many studies have shown a significant effect of clonal integration on morphological traits such as length and thickness of petioles and internodes of stolons and rhizomes (Alpert, 1999; Saitoh et al., 2002; Xu C. et al., 2012; Dong et al., 2015; Glover et al., 2015). Our results suggest that clonal epiphytes may not rely on integration-mediated changes in frond morphology to adapt to forest canopies.

## Conclusions

Our results indicate that clonal integration (resource sharing) may have been selected for as a general trait for clonal epiphytes to adapt to the harsh and heterogeneous epiphytic habitats. While epiphytes have been shown to take different strategies to adapt to forest canopies (Benzing, 2012; Lowman and Schowalter, 2012; Reyes-García et al., 2012), our study suggests that resource sharing is an additional one for clonal epiphytes. Epiphytes are a key component of forest canopies and play important roles in maintaining biodiversity (e.g., fauna diversity; Ozanne et al., 2003; Ellwood and Foster, 2004; May, 2010) and ecosystem functioning (e.g., carbon and nutrient cycling; Umana and Wanek, 2010; Benzing, 2012; Lowman and Schowalter, 2012). Considering that many epiphytes are clonal and also most of the dominant epiphytes are clonal (Jackson et al., 1985; During, 1990; de Kroon and van Groenendael, 1997; Benzing, 2012; Robinson and Miller, 2013), we hypothesize further that resource sharing may also play important roles during the underlying processes by promoting survival and growth of clonal epiphytes. Therefore, further studies could be designed to examine whether effects of resource sharing within clones of epiphytes can be cascaded to affect biodiversity and ecosystem functioning.

## Author contributions

WL and FY designed the project. HL, LS, and FY performed the experiments, analyzed the data and wrote the manuscript text. XX, YH, SL, ZF, and SGL analyzed some data and prepared some figures and tables. XS, WM, YC, and YW did some field work and collected data. All authors reviewed the manuscript.

### Conflict of interest statement

The authors declare that the research was conducted in the absence of any commercial or financial relationships that could be construed as a potential conflict of interest.
