# Supplementary material for: Survival and Growth of Epiphytic Ferns Depend on Resource Sharing
Source: Front Plant Sci. 2016 Mar 31;7:416. doi: 10.3389/fpls.2016.00416 (PMC4814527; doi:10.3389/fpls.2016.00416)
Supplement: Supplementary file 1 [file Table1.docx]

**Supplement Table 1** The eight fern species in dominant vascular epiphytes and their traits of rhizome or frond in the mountain moist forest in Ailao Mountain

| Dominant species | Rhizome | Frond (dry season) | References |
| --- | --- | --- | --- |
| *Polypodiodes subamoena* | Long and creeping | Withered | [Xu & Liu 2005](#_ENREF_71);  [Ma 2009](#_ENREF_40);  eFlora of China;  Personal observation. |
| *Lepisorus scolopendrium* | Thick, long-creeping | Withered |  |
| *Araiostegia perdurans* | Long and creeping | Withered |  |
| *Polypodiastrum argutum* | Long and creeping | Withered |  |
| *Vittaria flexuosa* | Creeping (short) | Overwintering |  |
| *Oleandra wallichii* | Long and creeping | Withered |  |
| *Polypodiodes microrhizoma* | Long and creeping | Withered |  |
| *Arthromeris lehmanni* | Long and creeping | Withered |  |
